# Crystal structure of c5321: a protective antigen present in uropathogenic *Escherichia coli* strains displaying an SLR fold

**DOI:** 10.1186/1472-6807-13-19

**Published:** 2013-10-07

**Authors:** Dunja Urosev, Mario Ferrer-Navarro, Ilaria Pastorello, Elena Cartocci, Lionel Costenaro, Dmitrijs Zhulenkovs, Jean-Didier Maréchal, Ainars Leonchiks, David Reverter, Laura Serino, Marco Soriani, Xavier Daura

**Affiliations:** 1 Institute of Biotechnology and Biomedicine, Universitat Autònoma de Barcelona, Bellaterra 08193, Spain; 2 Novartis Vaccines and Diagnostics Srl, Via Fiorentina 1, Siena 53100, Italy; 3 Department of Chemistry, Universitat Autònoma de Barcelona, Bellaterra 08193, Spain; 4 ASLA Biotech Ltd, Ratsupites 1, Riga 1067, Latvia; 5 Catalan Institution for Research and Advanced Studies (ICREA), Barcelona 08010, Spain

**Keywords:** c5321, Sel1-like repeat, Crystal structure, Super-helical fold, Antigen, Uropathogenic *Escherichia coli*

## Abstract

**Background:**

Increasing rates of antimicrobial resistance among uropathogens led, among other efforts, to the application of subtractive reverse vaccinology for the identification of antigens present in extraintestinal pathogenic *E. coli* (ExPEC) strains but absent or variable in non-pathogenic strains, in a quest for a broadly protective *Escherichia coli* vaccine. The protein coded by locus *c5321* from CFT073 *E. coli* was identified as one of nine potential vaccine candidates against ExPEC and was able to confer protection with an efficacy of 33% in a mouse model of sepsis. c5321 (known also as EsiB) lacks functional annotation and structurally belongs to the Sel1-like repeat (SLR) family. Herein, as part of the general characterization of this potential antigen, we have focused on its structural properties.

**Results:**

We report the 1.74 Å-resolution crystal structure of c5321 from CFT073 *E. coli* determined by Se-Met SAD phasing. The structure is composed of 11 SLR units in a topological organisation that highly resembles that found in HcpC from *Helicobacter pylori,* with the main difference residing in how the super-helical fold is stabilised. The stabilising effect of disulfide bridges in HcpC is replaced in c5321 by a strengthening of the inter-repeat hydrophobic core. A metal-ion binding site, uncharacteristic of SLR proteins, is detected between SLR units 3 and 4 in the region of the inter-repeat hydrophobic core. Crystal contacts are observed between the C-terminal tail of one molecule and the C-terminal amphipathic groove of a neighbouring one, resembling interactions between ligand and proteins containing tetratricopeptide-like repeats.

**Conclusions:**

The structure of antigen c5321 presents a mode of stabilization of the SLR fold different from that observed in close homologs of known structure. The location of the metal-ion binding site and the observed crystal contacts suggest a potential role in regulation of conformational flexibility and interaction with yet unidentified target proteins, respectively. These findings open new perspectives in both antigen design and for the identification of a functional role for this protective antigen.

## Background

CFT073 *Escherichia coli* is an uropathogenic strain responsible for conditions like cystitis and pyelonephritis (an ascending form reaching pelvis and kidneys), severe cases of which may lead to sepsis [1]. Uropathogenic *E. coli* (UPEC) bacteria are a subclass of ExPEC (Extraintestinal Pathogenic *E. coli*), a group of pathogens responsible for neonatal meningitis and septicaemia [2]. Increasing rates of antimicrobial resistance among uropathogens, complicating the future treatment of such infections, led to the development of vaccine preparations based on specific virulence factors, which unfortunately did not demonstrate long-term protection [3]. Hence, a broader approach to vaccine design, including the identification of non-virulence factors through methods such as immunoproteomics and reverse vaccinology (targeting of possible vaccine candidates starting from genomic information) is necessary. Recently, subtractive reverse vaccinology was used to identify a number of antigens present in ExPEC but absent or variable in non-pathogenic strains, suggesting that a broadly protective *E. coli* vaccine may be possible [2]. The 52 kD protein coded by locus *c5321* from CFT073 *E. coli* was identified as one of nine potential vaccine candidates against ExPEC and was able to confer protection with an efficacy of 33% in a sepsis mouse model [2]. Although an antibody-mediated response is likely to be responsible for the capacity of c5321 to induce protection in mice, the actual mechanism of action of anti-c5321 antibodies is still unknown. Recent data from our laboratories have suggested a role for c5321 in impairing the effector functions of human immunoglobulins indicating that antibodies directed against c5321 may affect the ability of *E. coli* to evade the immune system [4].

Sequence-based analysis performed with SMART [5] and PFAM [6] indicates that the protein is composed of Sel1-like repeats (SLRs, PFAM: PF08238). These repeats share a consensus sequence that is responsible for their helix-turn-helix (α/α) motif and are named after *Caenorhabditis elegans sel-1* gene product [7]. Such motifs are flexible in length, usually comprising 36–38 amino-acid residues, with few key positions of small and large hydrophobic residues. The crystal structure of the *Helicobacter pylori* cysteine-rich protein B (HcpB) [8], considered as a prototype of the structural fold consisting of SLR units, reveals the modular architecture with the α/α motifs arrayed in tandems and resulting in a super-helical fold. Structural domains composed of several such motifs are thought to act as interaction scaffolds to mediate protein-protein interactions. SLR units can be present in tandem arrays of up to 30 motifs or in groups dispersed throughout the protein sequence. SLR-containing proteins are found in both prokaryotic (more prevalent) and eukaryotic organisms, and are thought to have been acquired by horizontal gene transfer. Unfortunately, only few functional annotations are available for SLR proteins. There is accumulating evidence that *C. elegans* Sel1 is involved in degradation of proteins from the endoplasmic reticulum, while the yeast Hrd3 protein is thought to act as an adaptor protein for membrane-bound complexes and HcpA/B from *H. pylori* is speculated to be responsible for the adaptation of this bacterium to different hosts. It could be said that these molecular functions of SLR proteins are related, in that they are associated with signal transduction pathways [9].

SLR proteins share similar consensus sequence with the much more abundant TPR (tetratricopeptide repeat) protein family, in which TPR units are composed of 34 amino-acid residues [9,10]. The structural topology of TPR-containing proteins was revealed by the structure of the TPR domain of the protein phosphatase 5 (PP5) [11]. It displays a super-helical fold similar to the one characteristic of the SLR family. However, the superposition of this TPR domain with HcpB highlights different super-helix parameters, consequence of different packing angles within and between the repeats. The region of specific ligand binding, as observed in different TPR domains, is located in the amphipathic groove of the super-helix, with three tandem repeats likely being the optimal minimal length for binding [10,12]. Similar interactions most likely facilitate self-assembly into higher order structures [10,13]. TPR domains, as mediators of protein-protein interactions, have been implicated in a wide variety of cellular functions, such as transcription, cell cycle, protein translocation, protein degradation and host defence.

Due to the non-globular, rather elongated architecture of the repeating TPR and SLR units, where stabilization of the fold is achieved mostly through short-range interactions (along the primary sequence), the energy landscape of these proteins is distinct from that of globular proteins. Inter- and intra-element interactions of such quasi-one-dimensional structures are balanced in such a way that small local perturbations yield large effects, readily facilitating structural transitions that may be related to their biological function [14].

Unlike the case of TPR proteins, limited knowledge is available for SLR proteins, including fewer available crystal structures as well as functional annotations. Here, we report the 1.74 Å-resolution crystal structure of c5321 from CFT073 *E. coli* determined by Se-Met SAD phasing. The structure is composed of 11 SLR units, which to our knowledge represents the bacterial protein with the highest number of Sel1-like repeats solved up to date. It displays similar packing angles to those found in HcpB/C proteins from *H. pylori*, however with a distinct mode of overall fold stabilisation. Furthermore, we report the presence of a metal-ion binding site, generally uncharacteristic of TPR and SLR proteins. Crystal contacts between the C-terminal tail of c5321 and the C-terminal section of the amphipathic groove of a molecule belonging to the adjacent asymmetric unit are analysed and their possible biological relevance discussed. As part of a study of the antigenic properties of c5321, the regions of the protein that are recognised by antibodies have been mapped using murine monoclonal IgGs.

## Results and discussion

### Overall structure

The crystallographic structure of the functional unit (aa 24–490) of c5321 has been solved by SAD phasing and refined to a resolution of 1.74 Å. According to predictions by SignalP [15], the first 23 amino acids constitute a signal sequence. The final model has R and R_free_ values of 15.5% and 19.2%, respectively. The model has acceptable root-mean-square differences for bond lengths and angles (0.006 Å and 0.884 degrees, respectively) and none of the residues lie in disallowed regions of the Ramachandran plot (Table 1). One molecule is present in the asymmetric unit of the crystal. Amino-acid numbering in the model sequence reflects that of the functional unit (1–467 aa).

**Table 1:** Data collection and refinement statistics

		
**Data collection statistics**^**a**^		
Space group	P2_1_	
Type of crystal	Se-Met derivative (peak)	Se-Met derivative (peak)
Wavelength (Å)	0.9791	0.9794
Unit cell:		
a, b, c (Å)	48.94, 58.52, 88.35	
α, β, γ (°)	90.00, 103.82, 90.00	
Resolution (Å)^b^	37.91–1.74 (1.83–1.74)	58.57–2.28 (2.4–2.28)
Unique reflections	49642	22282
R_merge_ (%)	7.9 (48.4)	8.0 (25.4)
Completness (%)	99.5 (99.4)	99.7 (99.6)
Multiplicity	5.2 (5.2)	7.1 (7.1)
Average I/σ (*I*)	12.0 (3.1)	13.9 (5.7)
**Refinement statistics**		
Molecules/AU	1	
R (%)	15.5	
R_free_ (%)^c^	19.2	
B factor (Å^2^)	17.1	
RMS deviations:		
Bond lengths (Å)	0.006	
Bond angles (°)	0.884	
No. of:		
Protein residues	467	
Water molecules	466	
Hetero compounds	1 Mg^2+^, 2 Cl^-^, 29 ethylene glycol	
**Validation**		
Ramachandran plot:		
Favoured (%)	98.8	
Allowed (%)	1.2	
Disallowed (%)	0	

^a^Data collected at the ID14-4 MAD beamline of ESRF [35].

^b^Numbers in parentheses refer to the highest resolution shell.

^c^R_free_ was calculated using a subset (5%) of the reflections not used in the refinement.

**Figure 1:**
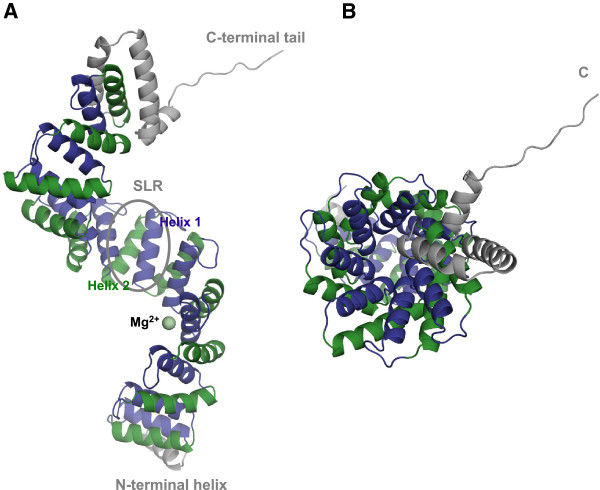
**Crystal structure of c5321 solved at 1.74 Å resolution. A**. Side view of the super-helix, with a Sel1-like-repeat (SLR) unit circled and the Mg^2+^-binding site between repeats 3 and 4 indicated in pale green. Helix-1 of the repeats is coloured in violet and helix-2 in green. N-terminal and C-terminal (likely capping) helices, as well as the C-terminal tail are represented in grey. **B**. Top-down view (along the vertical axis) from the C-terminus of the super-helix, depicting its concave (formed by the first helices of the repeats) and convex (formed by the second helices) surfaces.

c5321 displays a super-helical fold (Figure 1), containing eleven Sel1-like repeats, an N-terminal and two C-terminal helices with probable capping function and a (partly helical) C-terminal tail. Each repeat consists of two helices, helix-1 and helix-2, formed predominantly by thirteen residues, and connected predominantly by a 7-residue loop (Table 2). Individual repeats, tethered by three-residue loops, stack on top of each other creating an extended super-helical molecule with a continuous hydrophobic core. This structure can also be viewed as an overlapping array of three-helix bundles. The right-handed super-helix is approximately 115 Å in length, with a diameter of ~50 Å and a pitch (length of one complete helical turn measured parallel to the helix axes) of 60–65 Å. A complete helical turn comprises about seven to eight SLR units. The N- and C-terminal helices do not have a true SLR consensus sequence, but they share structural homology. A closer inspection of the amino-acid sequence suggests a role in ‘neutralizing’ hydrophobic surfaces on solvent exposed parts of the first and last repeats, hence facilitating the molecule’s solubility. To date, this is the known structure with the highest number of SLR repeats for a bacterial protein.

**Table 2:** Sel1-like repeat units in c5321

**SLR**	**Helix-1 region (#residues)**	**Intra-repeat loop #residues**	**Helix-2 region (#residues)**	**Inter-repeat loop #residues**
1	16–28 (13)	7	36–48 (13)	3
2	52–64 (13)	7	72–84 (13)	3
3	88–100 (13)	7	108–120 (13)	3
4	124–136 (13)	7	144–156 (13)	3
5	160–172 (13)	7	180–192 (13)	3
6	196–208 (13)	7	216–228 (13)	3
7	232–244 (13)	7	252–264 (13)	3
8	268–280 (13)	8	289–301 (13)	3
9	305–317 (13)	3	321–336 (16)	3
10	340–352 (13)	7	360–372 (13)	3
11	376–388 (13)	7	396–408 (13)	-

Uncharacteristic of SLR and TPR proteins, a metal-ion binding site, occupied by magnesium, was found between repeats 3 and 4 (Figure 1A). It resides in the negatively charged patch of the amphiphilic concave surface of the super-helix. Finally, the C-terminal region of the molecule contacts the C-terminal tail of the molecule in the adjacent asymmetric unit.

### Similarities with other SLR and TPR proteins

The packing angles of repeats are similar to those observed in the *H. pylori* cysteine-rich protein C (HcpC, PDB id 1OUV) [16], comprised of 267 residues (following signal-peptide cleavage) and sharing 47-53% sequence similarity (25-33% sequence identity) with c5321 (the highest structural homology with proteins in the PDB). An important difference between the two proteins resides in the inter-repeat disulfide bonds stabilizing the super-helical packing in HcpC, not present in c5321. Likewise, a shorter homologue (138 residues), *H. pylori* cysteine rich protein B (HcpB) (PDB id 1KLX) [8], has a 39-46% sequence similarity with c5321 and its repeats are also cross-linked by disulfide bonds. Finally, the putative Sel1-repeat protein kpn_04481 (228 residues) from *Klebsiella pneumoniae* ssp. *pneumoniae* (PDB id 3RJV, not published) shares 45-47% sequence similarity with c5321. While HcpC displays a pattern of repeat interactions similar to that found in c5321, with slight variation in packing angles within and between the repeats, kpn_04481 presents an uncommon packing angle of its fifth repeat that allows contacts with the intra-repeat loops belonging to the second and third repeats. Structure resolution and functional annotation of more SLR family proteins shall lead to understanding the necessity for this observed variety.

The first structure of a TPR-motif-containing protein was solved in 1998 [11] and, to date, the structure with the largest number of repeats (11.5) is that of the TPR domain of O-linked GlcNAc transferase (PDB id 1W3B) [17]. A distinct packing angle of the TPR units, as well as within repeats, allows a narrower super-helix in which, unlike SLR proteins, convex-face helical contacts are absent and those between concave helices are less extensive. The significance of these differences in packing between SLR and TPR assemblies is yet to be understood, but is likely related to distinguishing target proteins.

### Structural analysis of inter- and intra-SLR interactions

**Figure 2:**
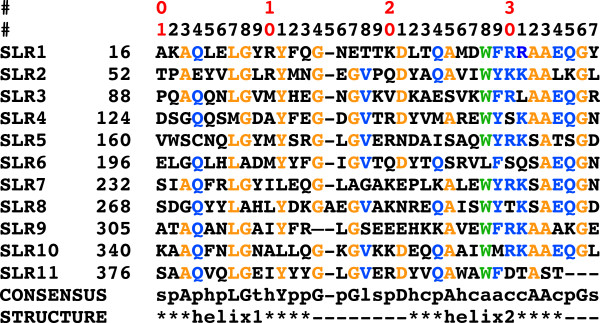
**Sequences of the eleven SLR repeats of c5321, aligned with the SLR consensus sequence.** First two rows provide a counter for intra-SLR position (first digit of the counter given in first row and second digit in second row). Second column corresponds to sequence number in full-length protein of first residue in the row. Specific amino-acid residues in the consensus sequence (from the SMART database [5]) are indicated with capital letters, while lower case letters stand for: p-polar; h-hydrophobic; t-turn like; s-small; c-charged; a-aromatic; l-leucine, valine or isoleucine. Secondary-structure elements are outlined in the last row, with loop regions being represented by hyphens (except SLR9, where helix2 is longer at the expense of the standard intra-repeat loop length). Residues matching specific conserved amino acids in the SLR consensus sequence are coloured in orange, while consensus amino-acid types with a dominant representative in c5321 SLR units are shown in blue (with the exception of W28r, which is coloured green for easy identification).

**Figure 3:**
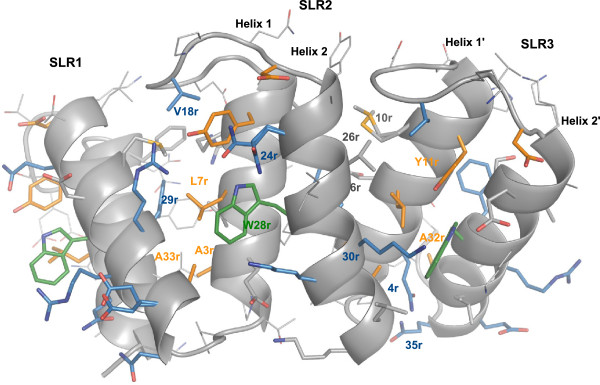
**Close-up view of the interactions within and between SLR repeats – convex face.** Residue numbers reflect intra-SLR positions as given in Figure 2. The amino-acid code (e.g. Y11r) is given when the specific amino acid is conserved in the position (as indicated in the consensus in Figure 2). Otherwise, only the position is specified (e.g. 24r). The colouring scheme is the same as in Figure 2. Residues in grey, with their side chains represented as sticks, reflect consensus areas with lack of dominant amino-acid representative. It does not necessarily reflect a reduced importance in packing.

**Figure 4:**
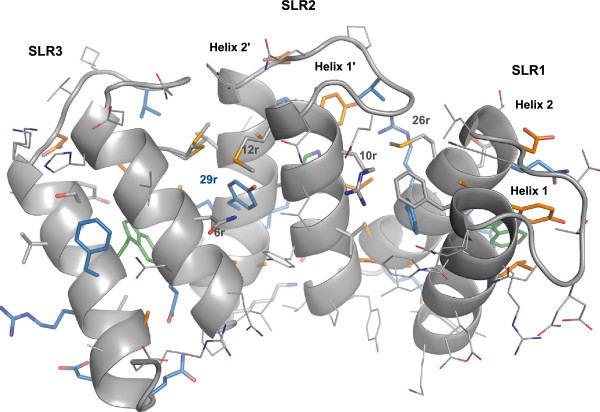
**Close-up view of the interactions within and between SLR repeats – concave face.** Residue numbers reflect intra-SLR positions as given in Figure 2. The colouring scheme is the same as in Figure 2. Concave face is of less-conserved character than convex face (Figure 3).

Even though the SLR family of proteins is known for a low conservation of the consensus sequence of the repeat, c5321 reproduces this sequence particularly well (Figure 2). Here, the definition of SLR derived from that of TPR, as annotated in the SMART database has been used. A repeat is composed of the more tightly packed helices, named helix-1 and helix-2 (Figures 1, 3 and 4). Alternatively, a repeat can be defined as constituted by the two helices packed at a wider angle (helix-2 and helix-1′), as suggested by Lüthy and collaborators based on the structures of HcpB and HcpC [8,16]. The rational for this alternative definition lies in the greater conservation of the latter repeat, reflected mainly in the constant length of the shorter loop between these helices. Herein we retain the SMART definition, as one could argue that the repeat should correspond to the entity containing the longer loop, which is an important component in defining the super-helical geometry.

The structure-based sequence alignment in Figure 2 shows that the majority of the 11 repeats retain particularly conserved amino-acid residues at positions 3, 7, 8, 11, 14, 17, 21, 25, 32, 33 and 36, indicated in orange, and conserved amino-acid types with dominant representative at positions 4, 18, 24, 28, 29, 30, 31, 34 and 35, coloured in blue. Position 28, occupied by aromatic residues in SLRs, is interestingly almost exclusively represented by tryptophan throughout the c5321 repeats.

The SLR consensus sequence highlights conserved glycine residues at positions 8, 14, 17 and 36, of which the last three facilitate turns in both intra- and inter-repeat loops while the first one, along with conserved alanine residues at positions 25 and 32, allows a close packing of the helices within the repeat. In turn, alanine residues at positions 3 and 33 favour the specific packing angle between repeats (Figure 3).

SLR proteins, unlike their TPR analogues, have few contacts across helix-2 units (convex-face helices). In c5321, they are mostly facilitated by the conserved tryptophan at position 28 (we shall use W28r to indicate repeat position), characteristic for this protein (Figure 2). Interestingly, c5321 helix-2 units have a slight kink, not found in HcpC, which effectively reduces the distance between these units at the side of the inter-repeat loop hence allowing the key positioning of W28r (Figure 3). On the other hand, existing interactions between helix-1 units (concave-face helices) tend to be less conserved (Figure 4). The angular geometry between repeats is mainly dictated by an inter-repeat hydrophobic core centred at conserved residue L7r in helix-1′ (Figure 3). L7r is in almost all cases in close contact with W28r in helix-2′, with residues 26r (hydrophobic or R), 29r (F or Y), 30r (primarily R) and A33r in helix-2 and with Y11r in the same helix-1′. Further contacts between helix-2 and helix-1′ include hydrophobic interactions between residues 10r and 26r, the interaction between residues 6r (polar) and 29r and those between the conserved Y11r and 30r, with the frequent presence of a hydrogen bond between the respective hydroxyl and guanidinium groups. Also, 30r (primarily R) is often hydrogen bonded via structural water to 24r (primarily Q) (not shown) (Figure 3). In the convex face, the inter-repeat hydrophobic core is protected from solvent by residues 26r and 30r from helix-2, Y11r from helix-1′, V18r from the intra-repeat loop and W28r from helix-2′.

Other conserved residue types (Figure 2) play an important role in intra-repeat interactions, such as hydrogen-bonded glutamine residues at positions 4 of helix-1 and 35 of helix-2, the stacking of Y11r and 24r (primarily Q), along with the already mentioned L7r and W28r contact (Figure 3).

Intra-repeat loops mainly consist of seven residues, with SLR8 containing one additional residue and SLR9 four residues less. Such relatively long loops are important for sufficient inter-repeat packing at wide angles, i.e. the length of these loops governs to a certain extent the stability of the inter-repeat geometry by capping the inter-repeat hydrophobic core. Loop tethering in the conformation observed in the structure is achieved mainly by interactions of conserved V18r with the inter-repeat hydrophobic core (26r and Y11r). The inter-repeat loop is shorter, three-residue long, and allows anti-parallel helical packing as well to limit the angle of inter-repeat helices to a certain degree (Figure 3).

Compared to its closest homologue HcpC, c5321 possesses greater sequence conservation and a different means for SLR-fold stabilisation. The Hcp family is unique among SLR proteins in that, in addition to the contributions from the constituent hydrophobic inter-repeat patch and the intra-repeat loop, fold stabilisation is achieved by disulfide bond tethering the C-terminal end of one repeat and the N-terminal end of the next repeat [8,16]. In the case of c5321, which displays a very similar inter-repeat angle, the disulfide-bond effect is most likely substituted by W28r-mediated interactions.

### Metal-ion binding site between SLRs 3 and 4

**Figure 5:**
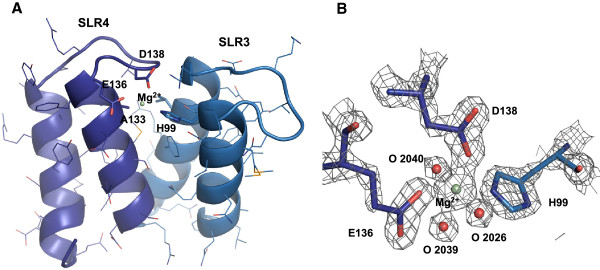
**Magnesium-ion-binding site in c5321. A**. Mg^2+^ coordination by residues H99 of SLR3, E136 of SLR4 and D138 of the intra-repeat loop. Alanine at repeat position 10 (A133) is indicated to highlight the lack of VdW contacts in the corresponding area. **B**. Electron density map (σ_A_-weighted 2Fo - Fc contoured at 2σ) at the metal-ion-binding site, including the three water molecules that complete the coordination.

Electron density corresponding to a magnesium ion is identified in the c5321 structure, with the metal-ion-binding site located between repeats SLR3 and SLR4. H99 in SLR3, E136 in SLR4 and its intra-repeat-loop residue D138, along with three water molecules, constitute the octahedral coordination ligands of the magnesium ion (Figure 5). Sequence alignment of SLR units does not reveal any other potential metal-ion-binding sites, of similar composition, in the protein.

Interestingly, the hydrophobic inter-repeat core is not as efficiently packed between repeats 3 and 4. This appears mainly due to alanine in position 10 of SLR4 (A133), occupied by larger residues in other repeats, and the corresponding loss of the interaction with positions 12 and 26 of SLR3 (Figure 5A). CASTp, an application for the detection of cavities in proteins [18], identifies a pocket between SLR3 and 4 (volume of 80.8 Å^3^, area of 95.6 Å^2^) as one of the top two in the SLR regions, where the other identified pocket is located between SLRs 8 and 9 that lacks the intra-repeat loop. In the case of SLRs 3–4, fewer important interactions between helix-1′ and helices 1 and 2 along with somewhat weaker intra-repeat loop tethering, in the absence of other compensatory mechanisms, would decrease the stability of the inter-repeat packing. Mg^2+^ coordination by residues of the intra-repeat loop, helix-1 and helix-1′ likely represents a means of stability/flexibility regulation in this region, for a currently unknown purpose. The significance of the intra-repeat-loop absence between SLRs 8 and 9, which could lead to a certain degree of instability in this region (due to greater solvent exposure of the hydrophobic core), is also unknown.

**Figure 6:**
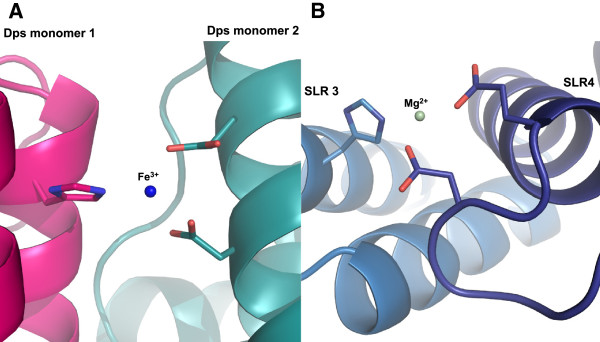
**Similarities between the Mg-ion binding site in c5321 and Fe-ion binding site in Dps proteins. A**. The Fe-ion binding site involving the triad His-Glu-Asp found in X-ray structures of Dps proteins (PDB ID 2FJC). **B**. Octahedral Mg-ion coordination observed in c5321 (water molecules not indicated).

As the likely origin of the magnesium ion in the c5321 structure is MgCl_2_ used in the crystallization conditions, the natural metal ion for this system and its binding specificities remain to be determined. Real-time quantitative PCR revealed that *c5321* mRNA levels in the uropathogenic strain CFT073 grown in Luria-Bertani (LB) medium were higher in the presence of specific ion chelators (including desferal and EDTA), suggesting a scenario where ions that are ligands to the protein are also regulators of its expression (Pastorello *et al.*, unpublished results). Unconventional iron binding, like the triad His-Glu-Asp involved in magnesium-ion coordination in the crystal structure of c5321, is found in X-ray structures of proteins closely related to ferritin and DNA-binding proteins from starved cells (Dps), e.g. Dps from *Mycobacterium smegmatis* (PDB id 1VEQ) or antigen TpF1 from *Treponema pallidum* (PDB id 2FJC). Interestingly, all these species are dodecameric entities presenting a spherical shell with a large inner cavity. The iron ion binds inside the cavity at the interface between two adjacent monomers. The histidine on one side, and glutamate and aspartate on the other, are provided by different subunits and configure the metal-binding site (Figure 6). In all Dps-like structures, the iron ion presents a possible tetrahedral or trigonal bipyramid geometry, given that water molecules involved in coordination might not be observed due to low resolution (e.g. 3.98-2.5 Å for the indicated structures). It cannot be excluded that the coordination of the metal-ion in c5321 could be of such lower order in a potentially native iron-bound structure.

Metal-ion binding, as evident from the PDB repository, is not common in SLR and TPR-containing proteins. However, an example where metal-ion binding might be partly responsible for dynamics and ligand-binding regulation is that of human Pex5p receptor. Sr^2+^ binding (physiological equivalent unknown) in the protein's TPR domain hinge region, even though in a coordination not resembling the case of c5321, leads to near rigid-body movement of its two halves (lobes) and less overall conformational flexibility of the domain [19].

Metal ions play a role in many important functions in proteins, including stability, conformational changes, folding and assembly. One can speculate that for c5321 the stabilisation of the SLR 3–4 region could represent a means of regulation of overall conformational flexibility, and in turn affect ligand (protein/peptide) binding (suspected to be in one of the major grooves, as discussed in the next section). Clearly, further investigation of metal-ion binding, its specificity and functional role will be required in order to assess these hypotheses.

### Crystal contacts between the C-terminal super-helical groove of one molecule and the C-terminal tail of the molecule belonging to the adjacent asymmetric unit

**Figure 7:**
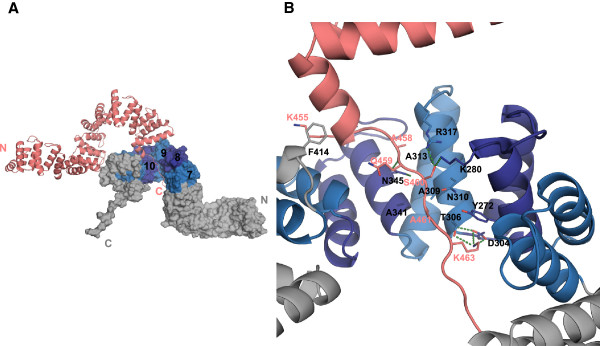
**Crystal contacts: binding of the C-terminal tail of c5321 to the C-terminal concave groove of a neighbour molecule. A.** Relative orientation of the two molecules, with SLRs 7–11 alternating in purple and light blue (the rest of the molecule is represented in grey). **B.** Close-up view of the interactions (see main text).

**Figure 8:**
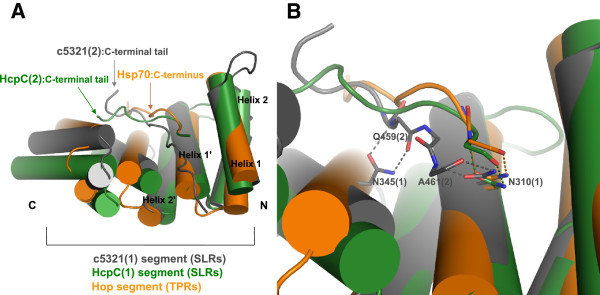
**Similarities between crystal contacts observed in c5321 and in HcpC and interactions of Hop with the C-terminus of its Hsp70 target (PDB ID 1ELW). A**. Structural superposition highlights similar mode of interaction involving concave region of the super-helix (molecule 1) and the extended peptide conformation of the C-terminus (molecule 2). **B**. Close-up of the hydrogen bonding interactions between the conserved asparagine residue (molecule 1, N310 in c5321) and the main chain of the C-terminus peptide (molecule 2). Note that the additional asparagine residue in c5321 (N345) is also involved in interactions with the main chain.

In the crystal packing the concave surface of the super-helix in the region of repeats 8, 9 and 10 of one molecule interacts with the C-terminal tail of a symmetry-related molecule (Figure 7A). Interestingly, similar C-terminal tail interactions have already been observed in other SLR proteins, such as HcpC (Figure 8).

Interactions are predominantly of polar character accompanied by van der Waals contacts. N345, belonging to repeat 10, participates in bidentate hydrogen-bonding interactions with the backbone carbonyl oxygen and nitrogen atoms of the C-terminal tail's Q459. N310 of repeat 9 is near the C-terminal tail's A461 and could be engaged in similar interactions with its backbone atoms, upon rotameric change (Figure 7B). These interactions can be categorised as anchoring, non-specific for peptide binding in such type of groove. Specificity might be governed by non-polar interactions of the tail peptide with the inter-repeat core and polar interactions with the solvent exposed repeat area. Thus, residues responsible for shape complementarity comprise A458 and A461 of the C-terminal tail in van der Waals contact with A313 and A341, A309, T306 of the second molecule, respectively. Polar interactions involve S460 hydrogen bonding to R317 in SLR9 and K280 in SLR8, while D304 and T306 in SLR9 take part in charge-charge and hydrogen-bond interactions with K463 in the C-terminal tail, stacking against Y272 of SLR8. Additional stacking interactions are observed between F414 belonging to one of the C-terminal helices and K455 at the C-terminal end (Figure 7B). The last three residues of the C-terminus, for which lower electron density is observed, do not really contribute to the interactions within this concave pocket.

Some similarity is shared with the mode of target-protein/peptide binding of TPR proteins, such as receptor for peroxisomal uptake-Pex5, Hsp70/Hsp90 organizing protein-Hop (Figure 8), FKBP52 and PP5. In these, TPR tandems recognise the C-terminal EEVD signal sequence of the target protein/peptide by a “carboxylate clamp” (group of conserved positively charged residues) [10,20-22] and likely also utilizing bidentate hydrogen-bonding interactions between conserved asparagine residues lining the super-helical groove and target peptide backbone atoms [17]. Figure 8 highlights this type of hydrogen bonding, involving a conserved asparagine residue, in c5321, HcpC and Hop/Hsp70(C-terminus). In most cases, the binding pocket establishing primary interactions through the target’s C-terminal tail is composed of three TPR repeats, in line with three tandem TPR domains being the most populated, suggesting that these represent the minimal functional binding unit. Secondary interactions are often important for establishing specificity and are thought to lie outside of this primary region, as shown for Hop [21,23]. Cortajarena *et al.* also emphasised the importance of both short-range interactions and long-range electrostatics as determinants of specificity [24].

On the other hand, there are examples of self association for TPR proteins, such as dimerisation of the Sgt1 plant protein [25] or oligomerisation of the MamA bacterial protein (demonstrated *in vivo)*, that involve their terminal helices binding in the super-helical groove regions [26]. For a number of TPR-containing proteins it has been shown that self-association can serve to regulate their biological function. SE-HPLC analysis of c5321 shows that aggregates/oligomers are present in very little amount (less than 5%, data not shown), although an exhaustive study of c5321 oligomerisation has not been performed.

The potential biological significance of the observed crystal contact in c5321 is revealed by comparison with the known TPR ligand-binding examples. Indeed, they share common features like binding in the concave area of the super-helix with similar peptide-backbone anchoring mode and three-tandem SLR domains as binding pocket. Correlation between the crystal contacts observed in the TPR protein Cyp40 and its interactions with the natural ligand (Hsp90) in solution [27] further suggests the possibility of a similar scenario in the c5321 case, with yet unidentified target protein/peptide (or self-association).

### Epitope mapping

**Figure 9:**
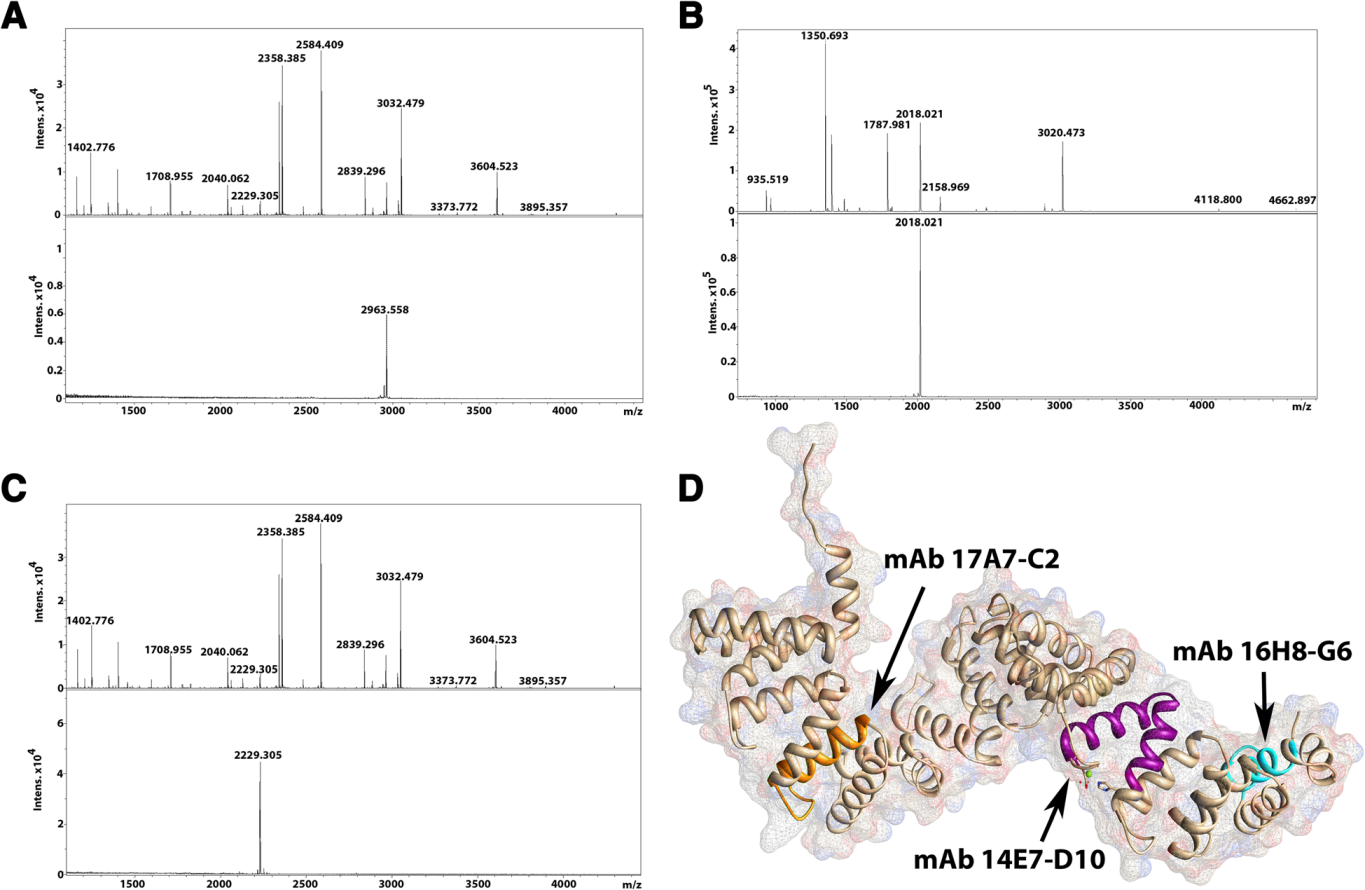
**Epitope-mapping results. A**. MS spectra of the proteolytic-digestion products of c5321 with GluC (upper spectrum) and the peptide immunocaptured with mAb14E7/D10 (111-SVKWFRLAAEQGRDSGQQSMGDAYFE-136, lower spectrum). **B**. MS spectra of the proteolytic-digestion products of c5321 with LysC (upper spectrum) and the peptide immunocaptured with mAb16H8/G6 (18-AQLELGYRYFQGNETTK-34, lower spectrum). **C**. MS spectra of the proteolytic-digestion products of c5321 with GluC (upper spectrum) and the peptide immunocaptured with mAb17A7-C2 (340-KAAQFNLGNALLQG-KGVKKDE-360, lower panel). **D**. Identified epitope-containing regions mapped onto the c5321 structure.

Mapping of c5321 epitopes for murine monoclonal antibodies was performed as part of the general characterization of this protein as a potential vaccine candidate, alongside presenting an opportunity to further investigate binding regions in c5321. Proteolytic digestion of the antigen following its incubation with monoclonal antibodies (see Methods) did not result in identification of any epitope-containing peptides. However, epitope-containing peptides were captured from partial digestion of c5321 (performed prior to incubation with monoclonal antibodies) with GluC (IgGs 17A7-C2 and 14E7/D10) or LysC (IgG 16H8/G6) (Figure 9).

The sequences of the epitope-containing peptides map to helix-1 of SLR1 for mAb16H8/G6, helix-1 of SLR10 for mAb17A7-C2 and helix-2 of SLR3 and helix-1 of SLR4 for mAb14E7/D10 (Figure 9D). Failure to immunocapture the products of LysC or trypsin cleavage (at the C-terminal side of arginine or lysine residues) by mAb17A7-C2 and mAb14E7/D10 further narrows down the important epitope components to the intra-repeat loop of SLR10 (containing three lysines) for mAb17A7-C2 and to the outer helix-2 of SLR3 (containing one lysine and two arginines) for mAb14E7/D10, respectively. These are in agreement with no steric hindrance to the access and binding of the antibody to these convex areas of the super-helix, and partly overlap with regions that are involved in Mg^2+^ binding (between SLRs 3 and 4) or belong to the C-terminal-tail binding groove (SLRs 8, 9 and 10). How relevant this observation is with respect to the previously discussed roles of these regions remains to be further investigated.

## Conclusions

We have solved the structure of c5321 from uropathogenic *Escherichia coli* to 1.74 Å resolution. This antigen displays a super-helical Sel1-like repeat fold with eleven SLR units and a remarkably preserved consensus repeat sequence. It shares high structural similarity with its closest homologue of known three-dimensional structure, HcpC from *Helicobacter pylori*, albeit with differences in how the SLR-fold is stabilized. While disulfide bridges in HcpC lock the characteristic inter-repeat geometry, in c5321 a conserved tryptophan residue at repeat position 28 appears to contribute fundamentally in maintaining the same geometry by strengthening the inter-repeat hydrophobic core. Metal ion binding, generally uncharacteristic of SLR proteins, is observed between SLR units 3 and 4, suggesting a regulatory role in conformational flexibility. Furthermore, crystal contacts observed between molecules belonging to neighbour asymmetric units share similarity to contacts characteristic for TPR-protein interactions with their physiological targets, suggesting a potential physiological interaction mode of c5321 with yet unidentified targets. The structure of c5321 is a first step for its functional characterisation and opens the door to the possibility of redesigning this antigen for vaccine-development purposes.

## Methods

### Cloning, expression and purification of c5321

*c5321* gene, without the predicted signal sequence, was amplified by PCR from the CFT073 genomic DNA template, cloned in pET-21b vector (Novagen) and transformed in DH5α-T1R chemically competent cells for propagation. BL21(DE3) chemically competent cells were used for His-tagged protein expression (6xHis at the C-terminus). Purification of the recombinant protein was performed from the bacterial soluble fraction using nickel-affinity chromatography as already described [28,29]. Cleavage of the His-tag was not performed.

### Expression and purification of selenomethionine-labelled c5321

The plasmid DNA containing the *c5321* gene downstream of the T7 promoter was transformed into B834 DE3 cells and the protein was expressed during 8 h at 25°C using the Overnight Express Autoinduction System 2 medium (Novagen) supplemented with 100 nM vitamin B12, 0.125 mg/ml selenomethionine and 50 μg/ml ampicillin. The cells were harvested by centrifugation at 5000 g for 30 min.

The cell pellet was suspended in lysis buffer (50 mM Na phosphate pH 7.5, 150 mM NaCl, 10 mM imidazole) and the cells were lysed by sonication. The insoluble fraction was removed by centrifugation (14000 g, 15 min) and the cleared lysate was applied onto a Ni-NTA sepharose column (Qiagen), equilibrated with the lysis buffer. The column was washed with 10 volumes of wash buffer (50 mM Na phosphate pH 7.5, 150 mM NaCl, 20 mM imidazole) and the protein was eluted with a gradient of increasing imidazole concentration (50 mM Na phosphate pH 7.5, 150 mM NaCl, 100 mM-300 mM imidazole). The protein-rich fractions were pooled and dialyzed 3 times against 100 volumes of 50 mM Na phosphate pH 7.5, 150 mM NaCl buffer. The protein sample was concentrated to 10 mg/ml using a 3 kD cut-off Amicon Ultra concentrator (Millipore). His-tag cleavage was not performed. The yield of the labelled c5321 was estimated to be about 20 mg of protein per litre of bacterial cell culture.

### Crystallisation and data collection

The protein was buffer exchanged with 5 mM Tris–HCl pH 8.0, 1 mM β-mercaptoethanol. Crystals were grown at 18°C using the hanging drop vapour diffusion method. The protein solution, at ~10 mg/ml concentration, was combined in a 1:1 ratio (v/v) with a well solution consisting of 20% PEG3350, 100 mM Tris–HCl pH 8.5, and 200 mM MgCl_2_. Prior to X-ray diffraction analysis, crystals were transferred to a cryo-protectant solution (20% ethylene glycol, 20% PEG3350, 100 mM Tris–HCl pH 8.5, and 200 mM MgCl_2_) and flash cooled in liquid nitrogen. Diffraction data were collected at 100 K at the beam-line ID14-4, ESRF, Grenoble. These data were indexed, integrated and scaled using MOSFLM and SCALA.

### Structure solution and refinement

The structure was solved by the Se-Met SAD method, using crystals that contained one molecule in the asymmetric unit. Se-Met SAD data at 2.28 Å resolution were collected to a satisfactory redundancy of 7.1 and relatively good signal/noise ratio of 13.9. Se atoms were located using Autosol, PHENIX [30] and initial phases were calculated from their positions. Model building was performed with the Autobuild module of PHENIX as well as Buccaneer [31], combined with manual reconstruction. Subsequent refinement was carried out with Phenix/Coot using a Se-Met SAD 1.74-Å resolution dataset and yielded an R factor of 15.5% and an R_free_ of 19.2%. Mg^2+^ was identified as the most likely representative for the electron density peak in proximity of repeats 3 and 4. 2 Cl^-^, 29 ethylene glycol and 466 water molecules were included in the final model. The quality of the final model was assessed with PROCHECK [32]. None of the residues lie in the disallowed region of the Ramachandran plot. The model was analysed using Pymol, which was used for figure preparation as well. Crystallographic statistics are shown in Table 1.

### Production of monoclonal antibodies against c5321

The purified recombinant c5321 was used to immunize CD1 mice. The first dose was a 50-μg injection, whereas the second and third doses at days 14 and 21 were of 25 μg. At day 28, anti-c5321 titres were measured in mice sera using ELISA plates coated with the recombinant c5321. A fourth dose was then administered, and 3 days later mice spleen cells were fused with myeloma cells (NS0). After 2 weeks of incubation in hypoxanthine-aminopterinthymidine selective medium, the hybridoma supernatants were screened for antibody-binding activity by ELISA. Hybridomas secreting anti-c5321 antibodies, selected by Western Blot to determine their capacity to recognize the antigen in bacterial extracts, were cloned by limiting dilution and then expanded and frozen for subsequent purification of mAbs. The mAb subclasses were determined using a mouse mAb isotyping kit (Roche). The mAbs were purified from culture supernatant by Protein G affinity columns (GE Healthcare), and after exhaustive dialysis in PBS buffer, the concentration of the purified mAb was determined by spectrophotometric reading at 280 nm.

### Epitope mapping of monoclonal antibodies

The epitope-mapping protocols are based on the approach described by [33], which we adapted to the two different protocols used here [34]:

1) *Immunocapturing of peptides from antigen partial digestion.* Peptide mixtures were obtained by digestion of c5321 with trypsin, LysC and GluC (separately) in 50 mM ammonium bicarbonate buffer in a ratio of 10:1 at 37°C for 3 h. To capture the epitope-containing peptide, a 25-μl suspension of Dyanbeads Pan Mouse IgG (uniform, super-paramagnetic polystyrene beads of 4.5 μm diameter coated with monoclonal human anti-mouse IgG antibodies) was used. The beads were washed twice with PBS using a magnet and re-suspended in the initial volume. 1 μg of the probe (murine) mAb was added and incubated for 30 min at room temperature, the beads were then washed twice with PBS to remove mAb excess. 0.5 μl of Protease Inhibitor Mix (GE Healthcare) was added before the peptide mixture to avoid potential degradation of the antibodies. The sample was incubated for 30 min at room temperature with gentle mixing. After incubation, the beads were washed three times with 1 ml PBS, and the bound peptide was then eluted with 50 μl of 0.2% TFA. The elute fraction was concentrated and washed with C18 ZipTips (Millipore) and eluted in 3 μl of 50% ACN and 0.1% TFA. For MALDI-MS analysis, 1 μl of sample was mixed with the same volume of a solution of alpha-cyano-4-hydroxy-transcinnamic acid matrix (0.3 mg/ml in H_2_O:ACN:TFA at 6:3:1), spotted onto the MALDI target plate and air-dried at room temperature. MALDI-mass spectra were recorded in the positive ion mode on an UltrafleXtreme MALDI TOF/TOF instrument (Bruker Daltonics). Ion acceleration was set to 25 kV. All mass spectra were externally calibrated using a standard peptide mixture. For MS/MS analysis, the MASCOT search engine (Matrix Science. London, UK) was used with the following parameters: one missed cleavage permission, 20-ppm measurement for MS and 0.3 Da for MS/MS tolerance. Positive identification were accepted with *p* < 0.05. In the searches, modification of methionine to methionine sulfoxide was allowed.

2) *Partial digestion of immunocaptured antigens.* To capture conformational epitopes, the order of the steps in the previous protocol was inverted. The intact protein (20 μg) was added to the beads, allowing it to bind to the immobilised mAb. The protease was then added to the sample in a ratio 50:1, and incubated at 37°C for 3 h. After proteolysis, the beads were washed ten times with 1 ml PBS, and the bound peptide was then eluted as previously described. To avoid the analysis of proteolysed antibody fragments within the elute fraction, c5321 was substituted by PBS in negative controls.

### Availability of supporting data

The coordinates and merged structure factors for c5321 have been deposited in the Protein Data Bank repository under accession code 4BWR [DOI:10.2210/pdb4bwr/pdb].

## Competing interests

The authors declare that they have no competing interests.

## Authors’ contributions

Designed the project: LS, MS, XD; designed and performed the experiments: DU, MFN, IP, EC, DZ; analysed the data: DU, MFN, LC, JDM, AL, DR, LS, MS, XD; wrote the manuscript: DU, MFN, LC, JDM, AL, LS, MS, XD. All authors read and approved the final manuscript.

## References

[B1] Welch RA Burland V Plunkett G 3rd Redford P Roesch P Rasko D Buckles EL Liou S-R Boutin A Hackett J Stroud D Mayhew GF Rose DJ Zhou S Schwartz DC Perna NT Mobley HLT Donnenberg MS Blattner FR Extensive mosaic structure revealed by the complete genome sequence of uropathogenic *Escherichia coli* Proc Natl Acad Sci USA 2002 99 17020 17024 10.1073/pnas.252529799 12471157 PMC139262

[B2] Moriel DG Bertoldi I Spagnuolo A Marchi S Rosini R Nesta B Pastorello I Corea VAM Torricelli G Cartocci E Savino S Scarselli M Dobrindt U Hacker J Tettelin H Tallon LJ Sullivan S Wieler LH Ewers C Pickard D Dougan G Fontana MR Rappuoli R Pizza M Serino L Identification of protective and broadly conserved vaccine antigens from the genome of extraintestinal pathogenic *Escherichia coli* Proc Natl Acad Sci USA 2010 107 9072 9077 10.1073/pnas.0915077107 20439758 PMC2889118

[B3] Hagan EC Mobley HLT Uropathogenic *Escherichia coli* outer membrane antigens expressed during urinary tract infection Infect Immun 2007 75 3941 3949 10.1128/IAI.00337-07 17517861 PMC1951972

[B4] Pastorello I Rossi Pacani S Rosini R Mattera R Ferrer Navarro M Urosev D Nesta B Lo Surdo P Del Vecchio M Rippa V Bertoldi I Gomes Moriel D Laarman AJ van Strijp JAG Daura X Pizza M Serino L Soriani M EsiB: a novel pathogenic E. coli SIgA-binding protein impairing neutrophil activation mBio 2013 4 e00206 e00213 23882011 10.1128/mBio.00206-13 PMC3735183

[B5] Schultz J Milpetz F Bork P Ponting CP SMART, a simple modular architecture research tool: identification of signaling domains Proc Natl Acad Sci USA 1998 95 5857 5864 10.1073/pnas.95.11.5857 9600884 PMC34487

[B6] Punta M Coggill PC Eberhardt RY Mistry J Tate J Boursnell C Pang N Forslund K Ceric G Clements J Heger A Holm L Sonnhammer ELL Eddy SR Bateman A Finn RD The Pfam protein families database Nucleic Acids Res 2012 40 D290 301 10.1093/nar/gkr1065 22127870 PMC3245129

[B7] Grant B Greenwald I The *Caenorhabditis elegans* sel-1 gene, a negative regulator of lin-12 and glp-1, encodes a predicted extracellular protein Genet 1996 143 237 247 10.1093/genetics/143.1.237 PMC1207257 8722778

[B8] Lüthy L Grütter MG Mittl PRE The crystal structure of Helicobacter pylori cysteine-rich protein B reveals a novel fold for a penicillin-binding protein J Biol Chem 2002 277 10187 10193 10.1074/jbc.M108993200 11777911

[B9] Mittl PRE Schneider-Brachert W Sel1-like repeat proteins in signal transduction Cell Signal 2007 19 20 31 10.1016/j.cellsig.2006.05.034 16870393

[B10] D’Andrea LD Regan L TPR proteins: the versatile helix Trends Biochem Sci 2003 28 655 662 10.1016/j.tibs.2003.10.007 14659697

[B11] Das AK Cohen PW Barford D The structure of the tetratricopeptide repeats of protein phosphatase 5: implications for TPR-mediated protein-protein interactions EMBO J 1998 17 1192 1199 10.1093/emboj/17.5.1192 9482716 PMC1170467

[B12] Allan RK Ratajczak T Versatile TPR domains accommodate different modes of target protein recognition and function Cell Stress Chaperones 2011 16 353 367 10.1007/s12192-010-0248-0 21153002 PMC3118826

[B13] Ramarao MK Bianchetta MJ Lanken J Cohen JB Role of rapsyn tetratricopeptide repeat and coiled-coil domains in self-association and nicotinic acetylcholine receptor clustering J Biol Chem 2001 276 7475 7483 10.1074/jbc.M009888200 11087759

[B14] Ferreiro DU Walczak AM Komives EA Wolynes PG The energy landscapes of repeat-containing proteins: topology, cooperativity, and the folding funnels of one-dimensional architectures PLoS Comput Biol 2008 4 e1000070 10.1371/journal.pcbi.1000070 18483553 PMC2366061

[B15] Petersen TN Brunak S von Heijne G Nielsen H SignalP 4.0: discriminating signal peptides from transmembrane regions Nat Methods 2011 8 785 786 10.1038/nmeth.1701 21959131

[B16] Lüthy L Grütter MG Mittl PRE The crystal structure of Helicobacter cysteine-rich protein C at 2.0 A resolution: similar peptide-binding sites in TPR and SEL1-like repeat proteins J Mol Biol 2004 340 829 841 10.1016/j.jmb.2004.04.055 15223324

[B17] Jínek M Rehwinkel J Lazarus BD Izaurralde E Hanover JA Conti E The superhelical TPR-repeat domain of O-linked GlcNAc transferase exhibits structural similarities to importin alpha Nat Struct Mol Biol 2004 11 1001 1007 10.1038/nsmb833 15361863

[B18] Liang J Woodward C Edelsbrunner H Anatomy of protein pockets and cavities: Measurement of binding site geometry and implications for ligand design Protein Sci 1998 7 1884 1897 10.1002/pro.5560070905 9761470 PMC2144175

[B19] Stanley WA Pursiainen NV Garman EF Juffer AH Wilmanns M Kursula P A previously unobserved conformation for the human Pex5p receptor suggests roles for intrinsic flexibility and rigid domain motions in ligand binding BMC Struct Biol 2007 7 24 10.1186/1472-6807-7-24 17428317 PMC1854907

[B20] Cliff MJ Harris R Barford D Ladbury JE Williams MA Conformational diversity in the TPR domain-mediated interaction of protein phosphatase 5 with Hsp90 Structure 2006 14 415 426 10.1016/j.str.2005.12.009 16531226

[B21] Onuoha SC Coulstock ET Grossmann JG Jackson SE Structural studies on the co-chaperone Hop and its complexes with Hsp90 J Mol Biol 2008 379 732 744 10.1016/j.jmb.2008.02.013 18485364

[B22] Alag R Bharatham N Dong A Hills T Harikishore A Widjaja AA Shochat SG Hui R Yoon HS Crystallographic structure of the tetratricopeptide repeat domain of Plasmodium falciparum FKBP35 and its molecular interaction with Hsp90 C-terminal pentapeptide Protein Sci 2009 18 2115 2124 10.1002/pro.226 19691130 PMC2786975

[B23] Ramsey AJ Russell LC Chinkers M C-terminal sequences of hsp70 and hsp90 as non-specific anchors for tetratricopeptide repeat (TPR) proteins Biochem J 2009 423 411 419 10.1042/BJ20090543 19689428 PMC3709441

[B24] Cortajarena AL Kajander T Pan W Cocco MJ Regan L Protein design to understand peptide ligand recognition by tetratricopeptide repeat proteins Protein Eng Des Sel 2004 17 399 409 10.1093/protein/gzh047 15166314

[B25] Nyarko A Mosbahi K Rowe AJ Leech A Boter M Shirasu K Kleanthous C TPR-Mediated self-association of plant SGT1 Biochem 2007 46 11331 11341 10.1021/bi700735t 17877371

[B26] Zeytuni N Ozyamak E Ben-Harush K Davidov G Levin M Gat Y Moyal T Brik A Komeili A Zarivach R Self-recognition mechanism of MamA, a magnetosome-associated TPR-containing protein, promotes complex assembly Proc Natl Acad Sci USA 2011 108 E480 487 10.1073/pnas.1103367108 21784982 PMC3158213

[B27] Taylor P Dornan J Carrello A Minchin RF Ratajczak T Walkinshaw MD Two structures of cyclophilin 40: folding and fidelity in the TPR domains Structure 2001 9 431 438 10.1016/S0969-2126(01)00603-7 11377203

[B28] Pizza M Scarlato V Masignani V Giuliani MM Aricò B Comanducci M Jennings GT Baldi L Bartolini E Capecchi B Galeotti CL Luzzi E Manetti R Marchetti E Mora M Nuti S Ratti G Santini L Savino S Scarselli M Storni E Zuo P Broeker M Hundt E Knapp B Blair E Mason T Tettelin H Hood DW Jeffries AC Identification of vaccine candidates against serogroup B meningococcus by whole-genome sequencing Sci 2000 287 1816 1820 10.1126/science.287.5459.1816 10710308

[B29] O’Hara AM Shanahan F The gut flora as a forgotten organ EMBO Rep 2006 7 688 693 10.1038/sj.embor.7400731 16819463 PMC1500832

[B30] Adams PD Afonine PV Bunkóczi G Chen VB Davis IW Echols N Headd JJ Hung L-W Kapral GJ Grosse-Kunstleve RW McCoy AJ Moriarty NW Oeffner R Read RJ Richardson DC Richardson JS Terwilliger TC Zwart PH PHENIX: a comprehensive Python-based system for macromolecular structure solution Acta Crystallogr D Biol Crystallogr 2010 66 213 221 10.1107/S0907444909052925 20124702 PMC2815670

[B31] Cowtan K The Buccaneer software for automated model building. 1. Tracing protein chains Acta Crystallogr D Biol Crystallogr 2006 62 1002 1011 10.1107/S0907444906022116 16929101

[B32] Laskowski R MacArthur M Moss D Thornton J PROCHECK: A Program to check the stereochemical quality of protein structures J Appl Crystallogr 1993 26 283 291 10.1107/S0021889892009944

[B33] Peter JF Tomer KB A general strategy for epitope mapping by direct MALDI-TOF mass spectrometry using secondary antibodies and cross-linking Anal Chem 2001 73 4012 4019 10.1021/ac010258n 11534730

[B34] Soriani M Petit P Grifantini R Petracca R Gancitano G Frigimelica E Nardelli F Garcia C Spinelli S Scarabelli G Fiorucci S Affentranger R Ferrer-Navarro M Zacharias M Colombo G Vuillard L Daura X Grandi G Exploiting antigenic diversity for vaccine design: the chlamydia ArtJ paradigm J Biol Chem 2010 285 30126 30138 10.1074/jbc.M110.118513 20592031 PMC2943275

[B35] McCarthy AA Brockhauser S Nurizzo D Theveneau P Mairs T Spruce D Guijarro M Lesourd M Ravelli RBG McSweeney S A decade of user operation on the macromolecular crystallography MAD beamline ID14-4 at the ESRF J Synchrotron Radiat 2009 16 Pt 6 803 812 19844017 10.1107/S0909049509035377 PMC2765085

